# Automatic Jordanian License Plate Detection and Recognition System Using Deep Learning Techniques

**DOI:** 10.3390/jimaging9100201

**Published:** 2023-09-28

**Authors:** Tharaa Aqaileh, Faisal Alkhateeb

**Affiliations:** 1 Department of Computer Science, Yarmouk University, Irbid 21163, Jordan; falkhateeb@uob.edu.bh; 2 Department of Computer Science, University of Bahrain, Sakheer 32038, Bahrain

**Keywords:** automatic license plate detection and recognition, automatic vehicle logo detection and recognition, deep learning, transfer learning, convolutional neural network

## Abstract

Recently, the number of vehicles on the road, especially in urban centres, has increased dramatically due to the increasing trend of individuals towards urbanisation. As a result, manual detection and recognition of vehicles (i.e., license plates and vehicle manufacturers) become an arduous task and beyond human capabilities. In this paper, we have developed a system using transfer learning-based deep learning (DL) techniques to identify Jordanian vehicles automatically. The YOLOv3 (You Only Look Once) model was re-trained using transfer learning to accomplish license plate detection, character recognition, and vehicle logo detection. In contrast, the VGG16 (Visual Geometry Group) model was re-trained to accomplish the vehicle logo recognition. To train and test these models, four datasets have been collected. The first dataset consists of 7035 Jordanian vehicle images, the second dataset consists of 7176 Jordanian license plates, and the third dataset consists of 8271 Jordanian vehicle images. These datasets have been used to train and test the YOLOv3 model for Jordanian license plate detection, character recognition, and vehicle logo detection. In comparison, the fourth dataset consists of 158,230 vehicle logo images used to train and test the VGG16 model for vehicle logo recognition. Text measures were used to evaluate the performance of our developed system. Moreover, the mean average precision (mAP) measure was used to assess the YOLOv3 model of the detection tasks (i.e., license plate detection and vehicle logo detection). For license plate detection, the precision, recall, F-measure, and mAP were 99.6%, 100%, 99.8%, and 99.9%, respectively. While for character recognition, the precision, recall, and F-measure were 100%, 99.9%, and 99.95%, respectively. The performance of the license plate recognition stage was evaluated by evaluating these two sub-stages as a sequence, where the precision, recall, and F-measure were 99.8%, 99.8%, and 99.8%, respectively. Furthermore, for vehicle logo detection, the precision, recall, F-measure, and mAP were 99%, 99.6%, 99.3%, and 99.1%, respectively, while for vehicle logo recognition, the precision, recall, and F-measure were 98%, 98%, and 98%, respectively. The performance of the vehicle logo recognition stage was evaluated by evaluating these two sub-stages as a sequence, where the precision, recall, and F-measure were 95.3%, 99.5%, and 97.4%, respectively.

## 1. Introduction

The automatic license plate and vehicle logo detection and recognition (ALP and VLDR) is an advanced computer vision-based task to identify vehicles without human intervention [1]. Moreover, the license plate (LP) is considered the identifier (ID) for each vehicle, which means that it is the primary information used to distinguish between vehicles [2]. Furthermore, the vehicle logo (VL) is used to complete vehicle identification information. Recently, the number of vehicles has increased dramatically on urban roads due to the increasing trend of urbanisation, so the ALP and VLDR systems are essential technology for intelligent transport systems (ITS), surveillance systems, and security. The main applications of ALP and VLDR in these systems are accident monitoring [3], detecting stolen vehicles [4], traffic control [5], vehicle control in autonomous vehicles and connected and automated vehicles (CAVs) [6,7,8,9], and so on.

Automatic license plate detection and recognition is sometimes known by various other terms such as Automatic number plate recognition or automatic license plate recognition. Moreover, the automatic vehicle logo detection and recognition task is known by another term, automatic vehicle manufacture detection and recognition. Recently, ALP and VLDR has become a vital topic and has attracted the attention of many researchers. However, they are still considered to be challenging tasks due to several reasons [10,11] such as the following:

- LPs Variations: There are many variations in LPs in terms of size, orientation, location, font, style, colour, language, etc.
- VLs Variations: There are many variations in VLs in terms of size, orientation, location, style, colour, shape, etc.
- Environmental Condition Variations: The vehicles are captured with different environmental conditions such as illumination (lighting), variance (contrast), shadows, etc.

In addition, many factors have made ALP and VLDR a challenging research topic, such as the dirt and oblique LPs and VLs, the quality of the camera that captures the vehicles, and the distance between the camera and the vehicles.

The structure of the LPs is determined by the government of the country to which the LP belongs. In Jordan, the LP consists of a maximum of seven digits where the first number or the first two numbers determine the use of the vehicle (code number(s)) while the remaining numbers determine the vehicle ID. Also, it contains the name of the kingdom in English and Arabic, which are “الاردن” and “JORDAN”, respectively. The numbers and letters of LP appear in black, and the background is in white. Further, the Jordanian LP consists of four colour codes: red, yellow, white, and green. In addition, two types of Jordanian LPs, American and European, differ in shape. American LP takes a square shape with 34∗22 cm size and consists of one or two numbers followed by the vehicle’s ID in a new line. European LP takes a rectangular shape 52∗11.4 cm in size and consists of one or two numbers followed by a dash symbol and the ID of the vehicle [12].

The UK Police Scientific Development Branch first invented the ALPDR tasks in 1976 [13], and then it became a hot research topic in most countries of the world. Typically, the system goes through several stages, and according to the previous research, several methods have been implemented for each stage as follows:

### 1.1. License Plate Recognition

This stage is responsible for recognising LPs from the input images as it passes through two other sub-stages to perform this task as follows:

#### 1.1.1. License Plate Detection

The methods that have been implemented to achieve this sub-stage can be divided into four main categories: traditional methods (i.e., image processing methods), hybrid image processing methods, machine learning (ML) methods, and deep learning (DL) methods.

In traditional license plate detection (LPD) methods, image processing techniques extract the most desirable features from the vehicle image. These features are used to detect LP as a region of interest (ROI), which is divided into two categories: handcraft features (inherent attributes) and general feature descriptors [14].

- Handcrafted Features—methods based on these features identify interesting features from the image as key points, called Feature Detector. Several features have been listed under this type, including boundary/edge [15] and colour [16].
- General Feature Descriptors—methods based on this type discover interesting features and describe the surrounding pixels called Feature Detector–Descriptor. These methods provide more information about the pixel area surrounding the key points, improving the performance of LPD. For example, the histogram of oriented gradients (HOG) [17].

In other cases, traditional methods have been hybridised to improve LPD accuracy. For example, a hybrid of colour and texture features [18].

Another trend is to use ML techniques along with image processing techniques, where ML techniques are used after extracting traditional features as classifiers. Once the traditional elements are extracted, an ML technique is trained on these features. After that, true LPs are extracted using ML techniques such as support vector machine (SVM) [19].

Recently, DL using the convolutional neural networks (CNNs) model has achieved remarkable results in accomplishing computer vision (CV) tasks, which include object detection and recognition [20]. Furthermore, CNN models have achieved the state-of-the-art (SOTA) performance for these tasks [21]. They are considered more efficient in dealing with complex and dynamic environments [6]. In addition, features are recognised using CNN models and extracted automatically [22]. So, several CNN models have been used for LPD tasks, such as Single-Shot MultiBox Detector (SSD) [23] and YOLOv2 [24].

#### 1.1.2. Character Recognition

For CR of LPs sub-stage, many methods have been used. These methods are classified into two categories: ML methods and DL methods.

1. ML methods include SVM [25].
2. DL methods include VGG-net [3] and SSD [26].

### 1.2. License Plate Recognition

This stage is responsible for recognising VLs from the input images as it passes through two other sub-stages to perform this task as follows:

#### 1.2.1. Vehicle Logo Detection

The methods that have been implemented to achieve the VLR stage can be divided into three main categories: traditional methods, ML methods, and DL methods.

1. Image processing methods: As mentioned earlier, the methods that fall under this category can be categorised into two types: traditional methods, such as perspective transformation [27] and general feature descriptors, such as Speeded-Up Robust Features [28].
2. ML methods include the AdaBoost classifier [29].
3. DL methods include ResNet-50 [30] and SSD [31].

#### 1.2.2. Vehicle Logo Recognition

The methods that have been implemented to achieve the VLR stage can be divided into four main categories: traditional methods, ML methods, and DL methods.

1. Traditional methods include HOG [32].
2. ML methods such as SVM [33].
3. DL methods such as LeNet-5 [34].

To our knowledge, the current works that have been applied for automatic Jordanian LP detection and recognition depend on traditional techniques (image processing techniques) [12,35,36]. However, these techniques only work under specific controlled conditions, detect and extract only local and low-level features, and suffer from the difficulty of manually designing discriminative features (labour intensive). So, the main goal of this research is to develop a system for identifying Jordanian vehicles by detecting and recognising the LP and logo of these vehicles. We apply DL-based CNN to accomplish the developed AJLP and VLDR system. In other words, all stages of the AJLP and VLDR system are mainly based on DL techniques that have achieved the SOTA performance in most CV tasks. Further, the current systems depend upon datasets, where the plate coordinates are given. Moreover, there is no available dataset for Jordanian vehicles, and existing VL datasets do not cover all VLs included in our research. So, we collected our datasets for training and testing the proposed system. This, in turn, allows automatic traffic control, detecting traffic violations, detecting stolen vehicles, etc.

The rest of the paper is organised as follows: Section 2 presents related work. Section 3 shows the research methodology. The collected dataset is presented in Section 4. Section 5 presents the results of evaluating its efficiency and discusses the findings. Finally, Section 6 presents the conclusions and future work.

## 2. Related Work

Over the last few decades, ALP and VLDR have become vital research topics and attracted the attention of many researchers. A lot of techniques can be found in the literature that have been used to achieve the stages of the ALP and VLDR system. In this section, we discuss the approaches that have been proposed for each stage in the ALP and VLDR system.

### 2.1. License Plate Recognition

This stage is responsible for recognising LPs from the input images as it passes through two other sub-stages to perform this task: LPD and CR. Several methods have been proposed for these sub-stages as follows:

#### 2.1.1. License Plate Detection

This sub-stage is responsible for detecting the LP’s place from the vehicle. Several methods have been used to achieve this stage successfully.

Generally, most LPs take a rectangular shape with a fixed aspect ratio, as image processing techniques can be used to detect all possible edges/borders (rectangles) of a vehicle image. Ref. [15] applied the Sobel edge detection operator after removing noise and undesirable features from the input images. The experiment was conducted on 300 Malaysian vehicle images and achieved an accuracy of 74.7%. Also, Ref. [37] applied a canny edge detection image processing technique after enhancing the images and removing the noises using histogram equalisation and median filtering, which slightly enhanced the detection process. The dataset used in this research consisted of 100 Chinese vehicle images.

Ref. [38] used canny edge detection along with Hough transform (HT) and Euclidean distance to detect the vertical and horizontal edges of Indian vehicles. However, this method produced poor results with extremely distorted images. HT technique cannot detect efficiently rotated images because it is very sensitive to rotation. In contrast, the connected component analysis (CCA) technique can deal with rotation. CCA is applied to binary images with 4- or 8-pixel neighbourhoods by scanning all pixels in the image, labelling them, and dividing them into blocks according to pixel connectivity (i.e., they have the same properties). For this reason, Ref. [12] applied an 8-pixel neighbourhood CCA technique with canny edge detection for 240 Jordanian vehicle images. Ref. [39] applied the CCA technique based on the aspect ratio of LP for 100 Bangladeshi vehicle images, as the accuracy was 93.78%. In [40], the improved Bernsen algorithm and CCA models were applied for three datasets: FZU cars, Stanford cars, and HumAIn 2019. Ref. [36] applied fast marching segmentation method to detect Jordanian LPs. The dataset used to conduct the experiment consisted of 100 vehicle images, where the accuracy was 95%.

Edge-based image processing techniques are usually easy to implement and have low time complexity but require clear, continuous edges to work efficiently. Morphological operations (MOs) such as opening, closing, dilation, erosion, etc., have been used to filter the image by removing undesirable edges. These operations greatly improved the detection process of LPs. In [10,41,42,43,44], edge detection with MO have been proposed. The datasets that were used were 100 Western Australia vehicle images, 100 Egyptian car images, 100 Indian vehicle front or rear images, 200 Indian LP images, and 40 four-wheel Myanmar vehicles, respectively. The accuracy of LP detection for this research was 92%, 78%, 89.5%, and 96.31%, respectively. The colour of LPs in each country is usually specific, so [16] applied HSI colour space with morphological open and close operations to localise Pakistan LPs. The dataset used consisted of 5543 images, where the accuracy was 96.72% accuracy.

Recently, DL technique-based CNN models have been introduced. These models achieved remarkable results in most CV tasks. Moreover, unlike ML techniques, DL techniques do not require a feature extraction step, as these features are automatically extracted without introducing hand-coded rules or human domain knowledge.

So Ref. [45], a sliding window-based selective search algorithm has been applied to generate all possible candidate areas that may contain an LP. Then, intersection over union overlap was applied to evaluate these candidate regions based on the ground truth bound/annotated bounding box. Finally, SVM was applied to make the final judgment (i.e., for classification). This method was examined on 1000 Chinese vehicle images and achieved 98.81% accuracy. Nevertheless, the selective search algorithm is slow and takes a long time to generate proposed regions. So, Ref. [46] adapted Faster Region CNN (R-CNN) based on the region proposal network (RPN) algorithm to generate the proposed regions for Chinese LPs. Two datasets were used to examine this method: the standard dataset and the real scene dataset, where the standard dataset contains 10,873 images and the real scene dataset contains 4711 images. In [47], the Mask R-CNN model was applied for LPD. This model can identify every pixel within the bounding box, improving the segmentation process accuracy. The experiment was conducted on 5000 Taiwanese LP images and achieved 91% accuracy. Ref. [48] mainly applied the Mask R-CNN model to detect Tunisian LPs. But the performance of this model was evaluated using four datasets, namely Caltech, AOLP, PKU, and Tunisian datasets, where the accuracy was 97.7%, 97.8%, 98.8%, and 97.4%, respectively.

Despite the fact that regional algorithms are accurate in detecting objects, they are very slow and have high computational costs. For these reasons, proposal-free algorithms have been proposed in the literature, directly detecting objects without generating any proposed regions. SSD has been used by [23], which is considered faster than Faster R-CNN. The experiment was conducted for Chinese LPs using 1700 low-resolution images and 3584 high-resolution images. Also, Ref. [26] applied SSD CNN for LP detection and achieved 98.3% accuracy for 50 K Chinese LPs. Ref. [24] applied the You Only Look Once (YOLOv2) model to detect Tunisian LPs. Two datasets were used for the experiment: GAP-LP, which consists of 9175 images and Radar datasets, which comprise 6448 images. The accuracy of the two datasets was GAP-LP 100% and 99.09%, respectively. Another proposal-free algorithm that has been applied is YOLOv3 by [49], which is considered a high-speed and real-time object detection algorithm. The dataset used consisted of 200 images of Bangla vehicles where the accuracy was 100% accuracy. Ref. [50] also applied YOLOv3 to detect Pakistani LP, which achieved an accuracy of 97.82% for 2131 vehicle images. In [51], the YOLOv3 model was applied to detect Iranian LPs, where the dataset used consisted of 5719 images, and the accuracy was 97.8%. Ref. [52] applied both CCA and the Inception-v3 model to detect the American LPs, where CCA was used to detect candidate regions and the Inception-v3 model was used to classify these regions. The dataset used to experiment consists of 126 Caltech cars, which achieved an accuracy of 96.72%. In [53], the SegNet model (semantic image segmentation) was applied to detect Northern Iraq LPs. The dataset used to conduct the experiments consists of 600 vehicle images, where the accuracy was 91.01%.

#### 2.1.2. Character Recognition

The last sub-stage of LP recognition is CR, which gives the result by recognising the characters of the LPs that have been segmented and extracted from the previous stage. Several methods have been proposed in the literature for this purpose.

Recently, DL-based CNN models have been used in most CV tasks, where they achieved state-of-the-art accuracy. Ref. [54] modified the Lenet-5 CNN model by removing the last convolutional layer in order to increase the processing time. The experiment was conducted on 112,000 US vehicles, where the accuracy was 93.001%. Ref. [26] modified SSD CNN model by removing the fully connected layer to increase the efficiency of CR. The efficiency of this model was measured through the experiment conducted on 90,213 Chinese LPs, where the accuracy was 99.1%. Ref. [3] applied Visual Geometry Group (VGGNet) model for Chinese vehicles. The experiment was conducted on 1403 vehicle images and achieved 97% accuracy. Ref. [49] applied YOLOv3 to recognise 200 Bangladesh vehicle images and achieved 99.5% accuracy. Ref. [50] also applied YOLOv3 to recognise Pakistani LP, which achieved an accuracy of 96% for 571 plate character images. In [51], the YOLOv3 model was applied to recognise Iranian LPs, where the dataset used consisted of 5719 images, and the accuracy was 95.05%. Ref. [55] applied a residual network with 18 layers (ResNet18) for Iranian vehicles. The experiment was conducted on two datasets: the IRCP dataset consists of 200 images, and the collected dataset consists of 350 images. The accuracy was 95% and 81%, respectively. In [56], the AlexNet CNN model was applied for feature extraction, and SVM was applied to classify these features. A multi-style dataset was used to evaluate this method, which consists of 3718 vehicle images from eight different countries, and the recognition accuracy was 96.04%. Ref. [52] applied the Inception-v3 model to recognise Taiwan LPs. The application-oriented license plate (AOLP) dataset consisting of 2049 images was used, where the accuracy was 98.9%.

### 2.2. Vehicle Logo Recognition

This stage recognises VLs from the input images as it passes through two other sub-stages to perform this task: VLD and VLR. Several methods have been proposed for these sub-stages as follows:

#### 2.2.1. Vehicle Logo Detection

This sub-stage is responsible for detecting the VL’s place from the vehicle. Several methods have been used to achieve this task successfully.

Ref. [57] applied overlapping enhanced patterns of oriented edge magnitudes method for VLD. The HFUT-VL dataset, consisting of 32,000 images belonging to 80 auto manufacturers was used, where the accuracy was 96.3%. Ref. [58] applied bottom-up visual saliency to locate the VL for 11,500 images belonging to 10 vehicle manufacturers, where the accuracy was 96.07%. Ref. [27] applied perspective transformation to detect 5 VLs with 2042 images.

As mentioned, all image processing techniques can achieve acceptable results under specific conditions. So, ML techniques have been used along with image processing techniques for detecting VLs. In [29], Haar-like features and the AdaBoost algorithm were applied to locate the VL from the input image. A dataset of 20,000 images belonging to eight vehicle manufacturers with an accuracy of 97% was used. Moreover, Ref. [59] applied the Adaboost-based learning method to detect the VL. A dataset of 1436 images belonging to 15 vehicle manufacturers with an accuracy of 93.94% was used. In [36], the VL is determined based on the locating of the LP using the fast-marching segmentation method. In other words, VL is detected based on prior knowledge that the VL is located on top of the LP. A dataset of 100 images belonging to five vehicle manufacturers was used.

Despite the robustness of ML techniques and their ability in object detection, it has a high computational cost due to the feature extraction step required to train an ML technique to apply the classification step based on these features. So, DL techniques have been used for detecting VLs. Ref. [60], applied the RPN to detect proposed regions that might contain VLs. A dataset of 225,000 images belonging to 15 vehicle manufacturers with an accuracy of 98.7% was used.

Even though regional algorithms are accurate in detecting objects, they are very slow and have high computational costs. For these reasons, proposal-free algorithms have been proposed in the literature, directly detecting objects without generating any proposed regions. In [11], the VGG16 and YOLOv2 model was applied, where VGG16 was used for feature extraction, and YOLOv2 was used for classification. A dataset of 2065 images belonging to 30 vehicle manufacturers was used. The experiment was conducted on single-scale and multi-scale images, where the accuracy was 74.4% and 78.5%, respectively. In [31], a multi-scale vehicle logo detector based on the SSD model was applied. The VLD-45 dataset, consisting of 45,000 images belonging to 45 auto manufacturers was used, where the accuracy was 84.8%, whereas [30] applied the ResNet-50 model to 8000 images belonging to 13 vehicle manufacturers, where the accuracy was 90.5%.

#### 2.2.2. Vehicle Logo Recognition

This sub-stage is responsible for recognising the detected VL from the previous sub-stage. Several methods have been used to achieve this task successfully.

Ref. [32] applied HOG descriptors with a feature selection method to recognise VLs. A dataset of 4000 images belonging to 40 vehicle manufacturers was used, where the accuracy was 75.25%. As mentioned earlier, all image processing techniques can achieve acceptable results under specific conditions. So, ML techniques have been used along with image processing techniques for recognising VLs. Ref. [33] applied scale-invariant feature transform descriptor with the SVM classifier, using 200 images belonging to five vehicle manufacturers, where the accuracy was 84.32%. However, this method fails to detect the true VL of images with low contrast and high occlusion. In [59], the SVM classifier with a pyramid of HOG and multi-scale block local ternary patterns features for VLR was used. A dataset of 1436 images belonging to 15 vehicle manufacturers was used, where the accuracy was 98.2%. In [61], a multi-feature fusion approach based on a two-level hierarchical classifier was applied. Three descriptors, namely, HOG, curvature histograms, and GIST, were used for feature extraction with the SVM classifier. Moreover, the grey wolf optimise method was used to improve recognition accuracy by optimising the kernel function. A dataset of 1072 images belonging to eight vehicle manufacturers was used, where the accuracy was 99.77%. In [57], a collaborative representation-based classifier was used for VLR. A dataset of 32,000 images belonging to 80 vehicle manufacturers was used, where the accuracy was 99.1%. Ref. [29] applied principal components analysis (PCA) to 20,000 images belonging to eight vehicle manufacturers, where the accuracy was 91%. In [62], grey level co-occurrence matrix PCA with K-nearest neighbour classifier was applied. A dataset of 1000 images belonging to 10 vehicle manufacturers was used, where the accuracy was 88.5%.

Despite the robustness of ML techniques and their ability in object detection, it has a high computational cost due to the feature extraction step required to train an ML technique to apply the classification step based on these features. So, DL techniques have been for recognising VLs. Ref. [34] applied the LeNet-5 model to identify VLs, using a dataset of 400 images belonging to 10 manufacturers, where the accuracy was 90.94%. Ref. [58] applied autoencoder pre-training deep neural network to identify VLs. The accuracy of 11,500 images belonging to 10 vehicle manufacturers was 99.20%. Ref. [27] applied Faster R-CNN and YOLOv2 models to 2042 images belonging to 5 classes. Ref. [63] applied the Faster R-CNN model with two CNN models, namely, VGG16 and ResNet-50, to 4000 images belonging to eight vehicle manufacturers. The accuracy was 94.33% and 87.71%, respectively. Ref. [30] applied multi-scale feature fusion YOLOv3 model to 8000 images belonging to 13 vehicle manufacturers.

As previously mentioned, vehicle control in autonomous vehicles and CAVs is one of the main applications of ITS. Therefore, it has recently attracted the attention of researchers. Ref. [9] proposed an integrated localisation method based on the fusion of an inertial dead reckoning model and 3D-LiDAR-based map matching. The vehicle’s initial heading and position were calculated using the developed vehicle-kinematics-based dead reckoning model based on sensory information of wheel speed and yaw rate. Then, a novel light frame generation method was used to refine the point cloud 3D-LiDAR. After that, the point cloud was transformed using the normal distribution transformation (NDT) based map matching algorithm. The final position and heading of the vehicle were obtained by calculating and correcting the error between position and heading from the dead reckoning model and the map matching algorithm. The performance of the proposed algorithm was tested on the ring road of the University of Waterloo under various environmental conditions (i.e., summer and winter). The experiments showed the effectiveness of the method in increasing the accuracy and reducing the computational complexity. Ref. [7] proposed a 3D-LiDAR-based cooperative perception that aims to improve object detection performance by leveraging historical object tracking data. This, in turn, reduced short-term occlusion and out-of-range problems. Moreover, a spatial–temporal deep neural network was proposed to extract the object tracking features. The V2XSet dataset was used to evaluate the performance of the method. The method achieved 0.7 average precision for object detection, which can also be generalised to object detection in a single vehicle with a 4.5% improvement in 0.7 average precision. In [8], an automated driving system data acquisition and analytics platform was proposed based on a cooperative perception of CAV to extract and reconstruct the vehicle trajectory. This platform can acquire and process sensor data from multiple CAVs and provides the object information in the map and Frenet coordinates, which includes object ID, position, speed, and orientation information. The Apollo CNN segmentation algorithm was used for object detection using LiDAR information with late fusion to fuse the detected objects from multiple CAVs. These detected objects are tracked by a multi-object tracking method to provide sensor information. Then, the off-road objects are filtered, and the down-track and cross-track information in the Frenet coordinate are obtained using the world model with HD. After that, reduce the noise in the trajectory using the Kalman filter and chi-square test method. Finally, detecting the discontinuity of the trajectory and reconstructing the trajectory using a fuzzy logic-based approach and a forward–backward smoothing technique, respectively.

According to the above literature review, and up to our knowledge, the works that have been applied for automatic Jordanian license plate detection and recognition depend on traditional techniques (image processing techniques). Furthermore, VLD and recognition methods do not cover all VLs included in our research. For these reasons and to benefit from the methods mentioned earlier advantages, we develop an AJLP and VLDR system based on DL-based CNNs using transfer learning (TL). The proposed system uses image processing techniques for image enhancement to increase our system’s accuracy in detecting and recognising LPs and VLs as an initial stage. Moreover, the DL-based CNN model will be re-trained using TL for LPs and VLs detection and recognition.

## 3. Methodology

In this research, we propose a system for automatically detecting and recognising Jordanian LPs and VLs using DL-based CNN models.

### 3.1. YOLOv3 Model

YOLOv3 stands for You Only Look Once, version 3, which was developed by Joseph Redmon and Ali Farhadi in 2018 from the University of Washington. As the name suggests, YOLOv3 passes the entire image only once (i.e., one forward propagation) through the network to make the prediction using 1∗1 convolutions. In other words, YOLOv3 simultaneously makes bounding box predictions and class probabilities. Therefore, it has been considered one of the most popular and fastest CNN models for real-time object detection. Generally, YOLOv3 passes through two stages to make the final prediction: feature extractor and feature detector [64].

#### 3.1.1. Feature Extractor

YOLOv3 uses a fully deep convolutional neural network as a backbone to extract appropriate features from the input images, namely Darknet-53. This network consists of 53 convolutional layers, each of which is followed by a batch normalisation layer and Leaky ReLU activation [64]. The normalisation layer’s main objective is stabilising the learning process of very deep CNN models by transforming and normalising the pixels (i.e., features) in the range 0 and 1. This enables the CNN model to converge faster [65].

In addition, in Darknet-53, a residual skip connection (residual block) was introduced as ResNet to solve the vanishing gradient and degradation problems caused by deep neural networks [66]. The residual block consists of consecutive 3∗3 and 1∗1 convolutional layers with a shortcut path [64,67].

Furthermore, Darknet-53 has no pooling layer; instead, a convolutional layer with stride two is used to downsample the feature maps in half. Therefore, Darknet-53 is referred to as a fully convolutional neural network. This helps prevent the loss of low-level features that are excluded by the pooling layer and improves the ability to detect small objects [64].

#### 3.1.2. Feature Detector

YOLOv3 uses another 53 layers stacked on the Darknet-53 convolutional layers for the detection task, resulting in 106 fully convolutional architecture layers for yolov3 [64].

Typically, other CNN models use the softmax function to determine the class of objects in the input image. Softmax assumes that classes are mutually exclusive; in other words, softmax assigns an object to one class with a high degree, whereas if an object belongs to one class, it cannot belong to another class [68]. However, the input image may include objects belonging to more than one class (label). Therefore, YOLOv3 performs multiple classifications of objects in the input image, where the softmax was replaced by a 1∗1 convolutional layer with independent logistic classifiers for each class with a predefined threshold to perform the classification (i.e., predict the class score for detected objects in the input image), where classes higher than this threshold are assigned to the predicted bounding box [64].

On the other hand, the input images may also include objects of different sizes. Other CNN models fail to detect small objects. Therefore, YOLOv3 uses a multi-scale feature pyramid network (MSFPN) to process different object sizes in the input image. MSFPN is placed in the YOLOv3 network as heads for feature extraction [64]. MSFPN includes two feature extraction paths: bottom-up path and top-down path. The bottom-up path is the usual convolutional network used to extract features (i.e., backbone). In contrast, the top-down path is used to create high-resolution features of a semantic-rich layer. Then, the top-down path features are enhanced with bottom-up path features via lateral connection. Each lateral connection merges feature maps with the same spatial size of the bottom-up and top-down paths. Simply, it merges high-level semantics with low-level semantics, leading to gathering more semantic features and preserving the fine features. This allows the YOLOv3 model to extract features of three different sizes at three different places in the network for prediction and to address the problem of small object detection [69].

#### 3.1.3. YOLOv3 Workflow

At first, YOLOv3 divides the input image into N grids with dimensions of S∗S. Each grid is responsible for detecting the object within it if the object centre belongs to that grid. As mentioned earlier, YOLOv3 applies a multi-scale detector using a feature pyramid network. This means that detection takes place at three different scales in three different places in the network to deal with large, medium, and small objects. So, after dividing the input image into grids, it passes to the stride layer to downsample its dimensions by 32, 16, and 8, respectively. This means that using an input image with dimensions of 416∗416, detection is made at the scales 13∗13, 26∗26 and 52∗52. Layer 13∗13 detects large objects, 26∗26 detects medium objects, and layer 52∗52 detects small objects [67,70]. The shape of the detection kernel used in each scale is $1*\left(B*\left(5+C\right)\right)$. Where B is the number of bounding boxes a cell on the feature map can predict, 5 represents the 4-bounding box attributes and one for object confidence, and C is the number of classes [71,72].

In addition, three scales were detected at three different places in the YOLOv3 network. In the first 81 layers, the image is downsampled by a stride of size 32, where the first detection is made by the 82 layers using the 1∗1 detection kernel. From the 79 layers, the dimensions of the feature map are upsampled by a factor of 2, which is then concatenated with the feature map of the same spatial size from the 61 layers. The second detection is made at the 94 layers with stride 16, where the feature map from the 91 layers is concatenated with the feature map of the same spatial size from the 36 layers. This feature map is then exposed to a few 1∗1 convolutional layers to merge the previous layer. The final detection is made at the 106 layers with stride 8 [71].

In YOLOv3, each grid in each scale can predict three bounding boxes using predefined anchors (priors). In total, nine anchor boxes are used, which are arranged in descending order, where the three largest anchors are assigned to the first scale, the next three to the second scale, and the last three to the third scale. Every predicted bounding box consists of width ($t_{w}$), height ($t_{h}$), centre ($t_{x}$,$t_{y}$), objectness score (confidence score) ($p_{0}$), and class scores (probabilities) ($p_{c}$). The confidence score checks if there is an object in the predicted bounding box. It is a probability between 0 and 1. Moreover, the confidence score is actually the intersection over the union (IoU) value between the prediction bounding box and the real box (ground truth). In general, the IoU is also known as the Jaccard Index, which describes how the bounding boxes overlap, where the predicted bounding box with an IoU greater than or equal threshold is taken as a positive prediction, otherwise, it is a negative prediction [73]. Class probabilities determine how likely a predicted bounding box is to belong to a particular class. Finally, these attributes are passed to a logistic classifier to determine each object’s class [64,74].

Since YOLOv3 divides the input images into grids, and each grid predicts three bounding boxes, the object can be predicted multiple times. Therefore, the non-maximum suppression technique is applied to select the most accurate bounding box and suppress any false detection [67,70].

### 3.2. VGG16 Model

VGG16 stands for Visual Geometry Group, also called OxfordNet. It is an off-the-shelf deep CNN model developed in 2015 by Karen Simonyan and Andrew Zisserman from the University of Oxford. The VGG16 model mainly comprises four parts: convolutional layers, pooling layers (max pooling layer), fully connected layers, and output layer (softmax layer). Fully connected layers and output layers are also called dense layers [75].

In the VGG16 model, convolutional layers have 3∗3 filters with a stride 1, which is used to extract the most appropriate features required to classify the objects in the input image. The max pooling layer has a 2∗2 filter with a stride of 2, which reduces computational cost and trainable network parameters by reducing the dimensions of feature maps obtained from convolutional layers. Fully connected layers are applied to previously obtained feature maps from pooling layers to flatten them as a 1-D matrix. This matrix represents the probability or class score vector. Finally, these flattened maps are passed to the softmax layer to determine the final decision about the class and its score for each object [75,76].

Basically, the VGG16 model starts with two repetitions of two convolutional layers followed by a max pooling layer. These layers are then followed by three repetitions of three convolutional layers, each followed by a max pooling layer. Finally, it consists of two fully connected layers followed by the classifier (softmax layer). The VGG16 model has thirteen convolutional layers, five max pooling layers, and two fully connected layers, followed by a softmax layer, giving us 21 layers. But VGG16 only has 16 layers with learnable weights, which are 13 convolutional layers and dense layers, so it was named VGG16 [76].

By default, the input of the VGG16 model is a fixed size of 224∗224 pixels with three channels of RGB images. Before passing the input images to the convolutional layers, they are subjected to a pre-processing step, which subtracts the mean image pixels (i.e., normalise the pixels) to be in the range 0 to 1 [75].

### 3.3. Research Methodology

**Figure 1:**
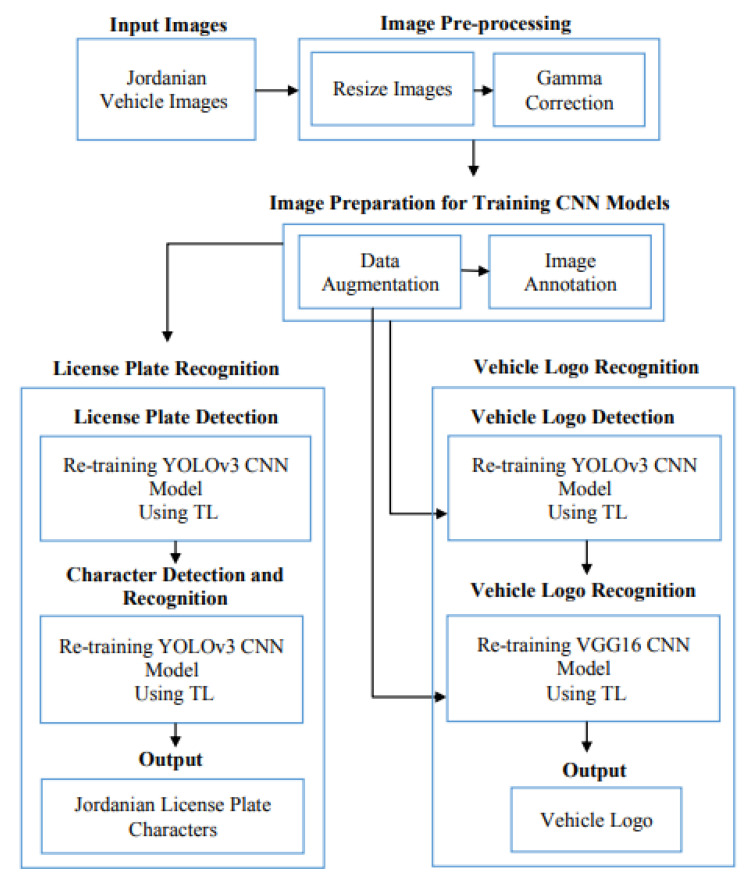
Proposed AJLP and VLDR system framework.

The proposed approach consists of three phases, as shown in Figure 1: image pre-processing techniques, dataset preparation, LPR using TL, and VLR using TL.

According to the above Figure, the proposed AJLP and VLDR system passes through several phases as follows:

1. Image Pre-processing:
  1.1. CNN models perform Image resizing automatically, where the image size of YOLOv3 and VGG16 is 416∗416 and 224∗224, respectively.
  1.2. Improve the brightness of the images using gamma correction.
2. Training phase steps for CNN models (i.e., YOLOv3 and VGG16):
  2.1. Dataset preparation:
    2.1.1. Use data augmentation techniques to increase the dataset size (i.e., the number of images).
    2.1.2. Annotating images of the YOLOv3 model using the LabelImg tool.
      For the LPR phase:
      LPD:
      Character detection and recognition:
        - The input is the Jordanian vehicle images with their corresponding annotation files.
        - Re-training the YOLOv3 model using TL.
      Character detection and recognition:
        - The input is the Jordanian LP images with their corresponding annotation files.
        - Re-training the YOLOv3 model using TL.
      For the VLR phase:
      VLD:
        - The input is the Jordanian vehicle images with their corresponding annotation files.
        - Re-training the YOLOv3 model using TL.
      VLR:
        - The input is the vehicle logo images.
        - Re-training VGG16 model using TL.
3. Testing phase steps of the proposed system (i.e., YOLOv3 model and VGG16 model):
  3.1. The input is the Jordanian vehicle image.
  3.2. Improving the input image as mentioned in step 1.
  3.3. The outputs are the LP characters and VL of the input image.

#### 3.3.1. Image Pre-Processing Techniques

The accuracy of the other stages of the AJLP and VLDR system depends mainly on the quality of the input images. Images are affected by several factors and conditions, the most important of which are environmental conditions such as illumination, variance, weather, etc. Several image processing techniques were used to improve the input images and clarify their details.

##### Resize Images

CNN models are affected by large-size images, which require too much memory and take a lot of computational time to process, train and test (output the result) [50,77]. Therefore, the Jordanian vehicle images were resized by automatically downsampling their dimensions by the YOLOv3 model while maintaining the same aspect ratio and keeping the LP and logo region predictable and recognisable. After resizing these images, their dimensions will be 416∗416 pixels. In addition, the VL images were resized by automatically downsampling their dimensions by the VGG16 model. After resizing these images, their dimensions will be 224∗224 pixels. As a result, the process will be sped up.

##### Gamma Correction

The input images’ brightness may affect the proposed system’s performance. Images may be captured in an unrestricted environment, and capture devices may not capture lighting/luminance properly. Moreover, the devices that display the input images may not display the original brightness correctly. Typically, the brightness/luminance of each pixel is a value between 0 and 1, where 0 indicates the darkness/black image, and 1 indicates the brightness/white image. The higher the gamma value, the higher the image brightness (the image tends to be white) and vice versa. The best value of gamma is 1.8 for outdoor images, which has been proven to achieve the best results [78]. Furthermore, this step was applied to all our datasets.

##### 3.3.2. Dataset Preparation

Two methods were used to prepare the datasets for the CNN model training process as follows:

##### Data Augmentation

Data augmentation (DA) is a technique used to improve the performance of the model being trained [48,79]. As mentioned earlier, a small dataset is not sufficient to build a more skilled and accurate DL model [80]. Therefore, DA is an option to automatically and artificially expand the size of the dataset [81]. The main objective of DA is to improve the model generalisation and avoid overfitting by increasing the quantity and diversity of the dataset [82]. Overfitting generally happens when a model is trained using a small dataset, where the features that are extracted from that dataset are not sufficient to be generalised to new data (unseen data) [65,81,82]. In our work, several realistic transformations were applied to our datasets as follows:

- Normalises images by 1/255 , which transforms image pixels between 0 and 1.
- Random rotation in range 0 and 45.
- Random zoom with a range equal to 0.2.
- Width and height shift with a range equal to 0.2.
- Random shear with a range equal to 0.2.
- Average blurring using a 5 ∗ 5 average kernel.
- Random translation with a range equal to 0.5.
- Random horizontal flip with a range equal to 0.5.
- Random crop with a range equal to 0.5.

These transformation methods have been applied to our datasets interchangeably. The first six transformations were applied to our VL dataset as they were all applied together for the first time. After that, these six methods were divided into groups, and each group was applied separately. Further, the last three transformations were applied by the YOLOv3 model automatically during the training process.

##### Image Annotation

As is known, object detection models require annotation files for training. Since YOLOv3 is considered an object detection model, text annotation files are needed. Typically, the annotation file contains information about the bounding box (ground truth) surrounding the object. LabelImg is a graphical annotation, free and open-source tool for manually annotating and labelling objects in images. This annotation tool is written in Python and uses Qt for developing the graphical interface. Moreover, it provides several annotation formats that many ML models support. These formats are VOC PASCAL for XML format and text files for YOLO [83,84].

Moreover, the name of each annotation file must be the same as the corresponding image name. In addition, annotation information about the bounding boxes of each object is saved in a text file for YOLO, each on a separate line. Each line has the following format [83]:

<object = class> <x-centre> <y-centre> <width> <height>

- <object = class>: It is a number ranging from 0 to (number of classes –1), which represents the class (label) of the object.
- <x-centre> and <y-centre>: x and y range from 0.0 to 1.0, representing the bounding box’s centre.
- <width> and <height>: width and height range from 0.0 to 1.0, representing the width and height of the bounding box.

#### 3.3.3. License Plate Recognition Using Transfer Learning

Object detection is a CV technique comprising two tasks: object detection (localisation) and object classification (recognition/identification). Object localisation is responsible for identifying objects’ location in the input image by drawing a bounding box around them, whereas object classification is responsible for classifying the localised object into a specific class [71,84,85]. In our work, the LPR stage is responsible for recognising the Jordanian LPs’ character from the input vehicle images. It passes into two other sub-stages to accomplish this task: LPD and CR.

##### License Plate Detection

Detecting the location of the LP from the input vehicle image is one of the most important stages because the accuracy of the CR stage depends on it. The YOLOv3 model is one of the most accurate and fast CNN models for object detection. This model was trained on a large-scale object detection dataset, Common Objects in Context, consisting of 330 K images belonging to 80 different classes (labels). The model achieved a mAP of 57.9% in 51 ms for object detection [64,66,86].

As it is known, TL is one of the most effective ways to improve training of DL CNN models on a small dataset since training such models from scratch requires a huge dataset [65]. Therefore, the YOLOv3 model was adapted to fit our task of Jordanian LPD using TL. As previously mentioned, the YOLOv3 model consists of two chained networks: the backbone (feature extractor) and the feature detector. The layers of the feature extractor network have no constraints on the number of filters (i.e., channel size/depth). In simple words, backbone layers have an arbitrary number of filters. In a feature detector network, the number of filters for all three detector layers (i.e., trainable layers) is constrained by the number of classes. Thus, if the number of classes changes, the number of filters (output size) in the detector network will change [64]. In our work, the number of categories for this sub-stage (Jordanian LPD) is one, which is the LP.

**Figure 2:**
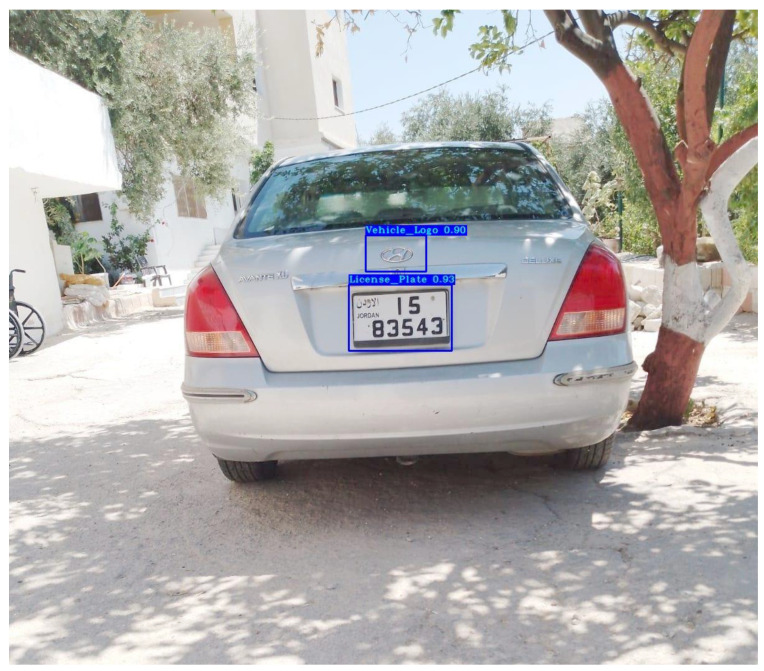
Outputs of the re-trained YOLOv3 model for the Jordanian LPD and VLD tasks. Note that the word “الاردن” is JORDAN.

Consequently, only trainable layers (i.e., detector layers) were re-trained on the labelled Jordanian LPs from our Jordanian vehicle dataset to fit the task of Jordanian LPD, while all other layers were frozen. The output of the fitted YOLOv3 model is the Jordanian LP location (the bounding box containing the LP) with the “License_Plate” label and its confidence score. Figure 2 shows the output of the re-trained YOLOv3 model for the Jordanian LPD task.

##### Character Detection and Recognition

**Figure 3:**
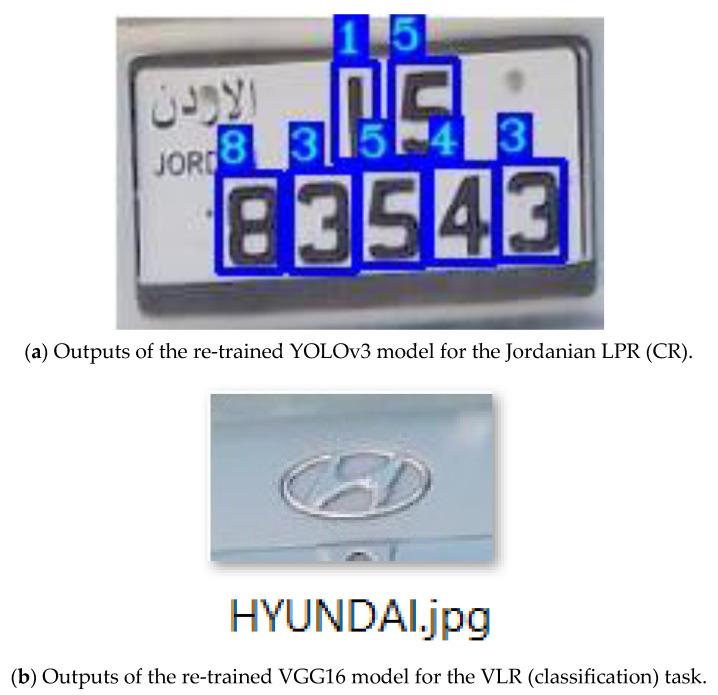
LPR and VLR stages outputs for the ALP and VLDR system. Note that the word “الاردن” is JORDAN.

This sub-stage is responsible for giving the result by recognising the characters from the previously detected Jordanian LP. Since the YOLOv3 model is very effective in detecting and recognising small objects, it has been re-trained to achieve this task using TL. In general, YOLOv3 has only three trainable layers (i.e., detector layers), constrained by the number of classes [64]. Since Jordanian LP consists of a maximum of seven characters (numbers), the number of classes is seven. Therefore, these trainable layers were re-trained on our dataset’s labelled Jordanian LP characters, and all other layers were frozen. Figure 3a shows the output of the re-trained YOLOv3 model for the Jordanian LPR (character) task of the previously detected LPs presented in Figure 2.

#### 3.3.4. Vehicle Logo Recognition Using Transfer Learning

In this stage, the vehicle manufacturer (i.e., VL) is recognised from the input vehicle images. To achieve this task, this stage goes through two other sub-stages: VLD and VLR.

##### Vehicle Logo Detection

This sub-stage is responsible for detecting the logo from the input vehicle images. As mentioned earlier, the YOLOv3 model outperforms other object detection models in detecting small objects [64]. So, it was fitted to achieve the VLD task by re-training its trainable layers (i.e., detector layers) on the labelled VLs from our Jordanian vehicle dataset and all other layers were frozen. The output of the fitted YOLOv3 model is the VL location (i.e., the bounding box containing the VL) with the “Vehicle_Logo” label and its confidence score. Figure 2 shows the output of the re-trained YOLOv3 model for the VLD task.

##### Vehicle Logo Recognition (Classification)

VLR (i.e., vehicle logo classification) is the last stage of our developed system, which is responsible for recognising (classifying) the previously detected logo. VGG16 model is considered an off-the-shelf classification model that achieved 92.7% accuracy in ImageNet dataset, consisting of fourteen million images belonging to a thousand different classes [75]. Since the domain and task of the VGG16 model are similar to our domain and task, we used them as a starting point for solving our task. In other words, we used the VGG16 model as a feature extraction and then added and re-trained our dense layers (i.e., fully connected layers and classification layer).

The process of re-training the VGG16 model using TL to fit the task of recognising the VL (classification) is as follows:

1. At first, we instantiated a base model and then loaded the weights of the pre-trained VGG16 base layers (backbone/feature extractor) onto it.
2. Dense layers have been cropped, leaving only the base layers (i.e., convolutional and pooling layers) by setting include_top = false.
3. Layers in the instantiated base model have been frozen by setting trainable = false to avoid destroying any features (weights) during training rounds.
4. New trainable layers (dense layers) have been added on top of the frozen layers and have been re-trained on our VL dataset to fit the VLR task.
5. Dropout layers have been added after the activation function (fully connected layers) to reduce the overfitting.

Figure 3b shows the output of the re-trained YOLOv3 model for the VLR (classification) task of the previously detected VLs presented in Figure 2.

## 4. Data Sample

In this work, we collected four different datasets to accomplish the AJLP and VLDR system (i.e., to train and test CNN models). To our knowledge, there is no publicly available dataset for Jordanian vehicles. So, we collected our own dataset by taking photos of Jordanian vehicles from different places: garages, public streets, malls, universities, and hospitals. The vehicle images were captured from front and rear at different angles from −35° to 35° at various distances from 2 m to 8 m using a Canon digital camera. Moreover, these images were captured in different illumination conditions and environments (i.e., day, sunny, and cloudy). The total number of captured images is 8271 with 6000 × 4000 resolution and JPG format. The collected dataset consists of the following attributes:

- A total of 7102 single vehicles.
- A total of 1169 multiple vehicles.
- A total of 2290 front vehicles.
- A total of 7645 rear vehicles.
- A total of 7265 vehicles were captured in sunny weather.
- A total of 1006 vehicles were captured in cloudy weather.
- A total of 8271 vehicles were captured during the day.
- A total of 3853 vehicles with American LPs.
- A total of 6082 vehicles with European LPs.
- LPs colour codes:
  1. A total of 9838 vehicles with white colour codes.
  2. A total of 81 vehicles with green colour codes.
  3. A total of 8 vehicles with yellow colour codes.
  4. A total of 8 vehicles with red colour codes.

As mentioned earlier, the LPR stage consists of two other sub-stages: LPD and CR. Therefore, two different datasets were used to accomplish this stage. For the first sub-stage (i.e., LPD), we used a dataset consisting of 7035 Jordanian vehicle images taken from the images we captured, whereas, for the second sub-stage (i.e., CR), we manually cropped Jordanian LPs from the vehicle images used in the first sub-stage. In total, the number of cropped Jordanian LPs was 7176. As a result, these cropped LPs formed a new dataset.

Furthermore, two other different datasets were used to accomplish our developed system’s second stage (i.e., VLR). This stage consists of two other sub-stages: VLD and VLR. For the first sub-stage (i.e., VLD), we used a dataset of all 8271 Jordanian vehicle images we captured, whereas, for the second sub-stage (i.e., VLR), we collected 152,572 VL images. These images were collected as follows:

- We cropped some of these images from the Jordanian vehicles images we captured, which equals 11,147 images.
- We collected some of them from different websites, which equals 36,620 images.
- We generated some of them by applying DA techniques, which equals 104,805 images.

In addition, we used some images from the VL dataset created by Kuba Siekierzynski after filtering, which equals 5658 images belonging to 28 VLs [87]. Eventually, the dataset used for the VL recognition sub-stage consists of 158,230 images distributed over 39 VL classes, which covers all the logos in the Jordanian vehicle images we captured. These classes are AUDI, BMW, BRENTHON, BUICK, C_EMBLEM, CHEVROLET, CITROEN, DAEWOO, DAIHATSU, DODGE, FIAT, FORD, GENESIS, GMC, GT_EMBLEM¸ HONDA, HYUNDAI, ISUZU, JEEP, KIA, LANCIA, LAND_ROVER, LEXUS, LINCOLN, M_EMBLEM, MAZDA, MERCEDES_BENZ, MERCURY, MITSUBISHI, NISSAN, OPEL, PEUGEOT, PORSCHE, RENAULT, RENAULT_SAMSUNG, SUZUKI, TESLA, TOYOTA, and VOLKSWAGEN.

## 5. Results and Discussion

In this section, we present and discuss the experimental results which were carried out based on the proposed methodology.

### 5.1. Data Sample Partitioning

The previously mentioned datasets used to accomplish the developed AJLP and VLDR system were divided into three partitions (i.e., training, validation, and testing) with specific ratios, as shown in Table 1.

### 5.2. System Validation

In this sub-section, we conducted experiments to validate the performance of our developed AJLP and VLDR system. The implementation (training) and experiments were carried out on an Intel (R) Xeon ^®^, 2.79 GHs CPU, 32 GB RAM, and 64-bit Windows 10 operating system with NVIDIA Tesla k40 GPU using Python programming language with TensorFlow and Keras libraries. Moreover, all the CNN models’ input sizes and hyper-parameters were configured, as shown in Table 2, to perform the training (i.e., TL).

The performance of the CNN models used to achieve the developed system was monitored and examined during the training process using learning curves. Learning curves belong to two types: optimisation learning curves and performance learning curves. Optimisation learning curves are charts that are calculated using optimisation matrices, by which model parameters are optimised, such as loss. Performance learning curves are also charts that are calculated using performance metrics, such as accuracy, by which the model is evaluated and selected. Furthermore, these curves are calculated for both the training and validation datasets during the training process. Thus, two curves are generated: the train learning curve and the validation learning curve. The training learning curve is calculated on the training dataset, giving an idea of how well the model has learned, whereas the validation learning curve is calculated on the validation dataset, which gives an idea of how good the model is to generalise [88]. In our work, the optimisation learning curve was calculated for the YOLOv3 model, while the performance learning curve was calculated for the VGG16 model as follows:

#### 5.2.1. License Plate Recognition

During the YOLOv3 training process, the optimisation learning curve is calculated using the loss function for both the training and validation datasets in each batch. As a result, two charts are generated: training loss and validation loss. The training loss chart shows how well the model fits the training dataset, while the validation loss chart gives an idea of how well the model fits the unseen dataset (new dataset). The lower the loss, the better the model’s performance [89]. In our work, training loss and validation loss were stored in a log file during the training process and then visualised using the TensorBoard provided with TensorFlow. Generally, the YOLOv3 loss function is the sum of box regression loss (coordinates loss), confidence loss (objectness loss), and classification loss [72].

##### License Plate Detection

**Figure 4:**
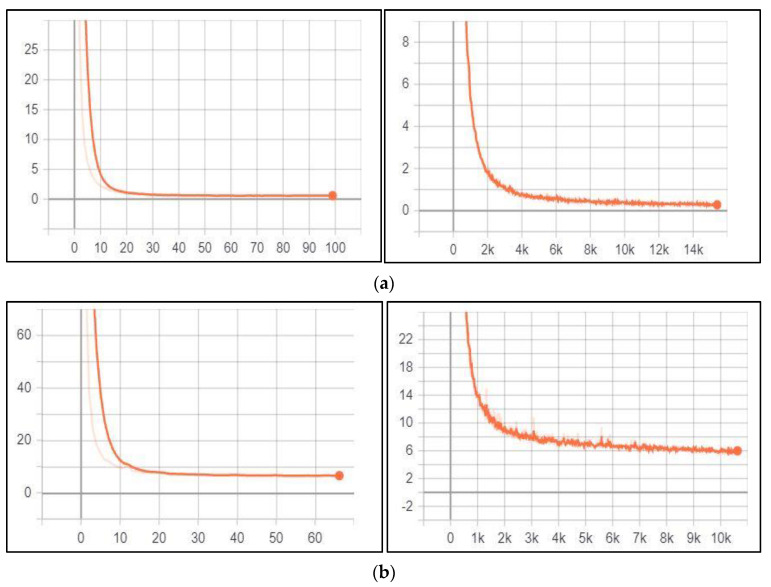
Optimisation learning curves for LPD and character detection and recognition stages for the ALP and VLDR system. (**a**) Optimisation learning curves for LPD stage. (**Left**): YOLOv3 total training loss for LPD task, where the *x*-axis represents the total loss, and the *y*-axis represents the number of steps. (**Right**): YOLOv3 total validation loss for LPD task, where the *x*-axis represents the total loss, and the *y*-axis represents the number of epochs. (**b**) Optimisation learning curves for character detection and recognition stage. (**Left**): YOLOv3 total training loss for character detection and recognition task, where the *x*-axis represents the total loss, and the *y*-axis represents the number of steps. (**Right**): YOLOv3 total validation loss for character detection and recognition task, where the *x*-axis represents the total loss, and the *y*-axis represents the number of epochs.

While training the YOLOv3 model on the Jordanian LPD task, its performance was monitored and validated using the loss function. Figure 4a illustrates the training and validation loss for this task, respectively.

According to the above figure, both training loss and validation loss gradually decrease, which tend to coincide after epoch 40. The training loss was 0.24, and the validation loss was 0.60. Moreover, the training loss indicates that the model fits the training dataset, converges rapidly after epoch 40 and remains stable. Also, the validation loss indicates that the model fits the new dataset (i.e., the model is generalisable).

##### Character Detection and Recognition

The performance of the YOLOv3 model for the character detection and recognition task was monitored and validated during the training process using the loss function. Figure 4b illustrates this task’s training loss and validation loss, respectively.

According to Figure 4b, training and validation loss gradually decrease, which tend to coincide after epoch 50. The training loss was 6.086, and the validation loss was 6.745. Moreover, the training loss indicates that the model fits the training dataset, converges rapidly after epoch 50, and remains stable. Also, the validation loss indicates that the model fits the new dataset (i.e., the model is generalisable). Initially, we set the training epochs to 100, but the model stopped improving (i.e., converged) after the epoch 67. So, we stopped the training process in this epoch.

#### 5.2.2. Vehicle Logo Recognition

In this stage, the performance was monitored and validated using both learning curves mentioned earlier. The optimisation learning curve was used for the YOLOv3 model dedicated to VLD tasks, which is the loss, whereas, for the VGG16 model dedicated to VLR (classification), the performance learning curve was used, which is the accuracy.

##### Vehicle Logo Detection

**Figure 5:**
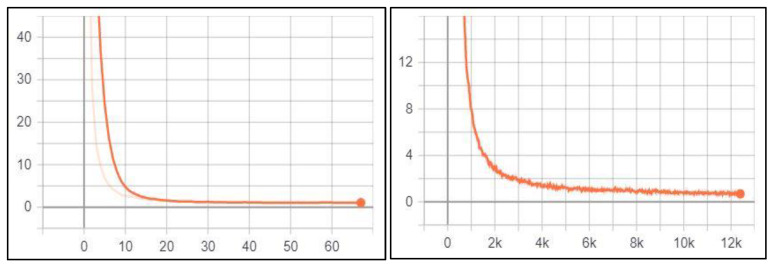
Optimisation learning curves for VLD stage. (**Left**): YOLOv3 total training loss for VLD task, where the *x*-axis represents the total loss, and the *y*-axis represents the number of steps. (**Right**): YOLOv3 total validation loss for LPD task, where the *x*-axis represents the total loss, and the *y*-axis represents the number of epochs.

The performance of the YOLOv3 model for the VLD tasks was monitored and validated during the training process using the loss function. Figure 5 illustrates the training loss and the validation loss for this task, respectively.

According to the above figure, both training loss and validation loss gradually decrease, which tend to coincide after epoch 50. The training loss was 0.726, and the validation loss was 1.002. Moreover, the training loss indicates that the model fits the training dataset, converges rapidly after epoch 50 and remains stable. Also, the validation loss indicates that the model fits the new dataset (i.e., the model is generalisable). Initially, we set the training epochs to 100, but the model stopped improving (i.e., converged) after epoch 68. So, we stopped the training process in this epoch.

##### Vehicle Logo Recognition (Classification)

**Figure 6:**
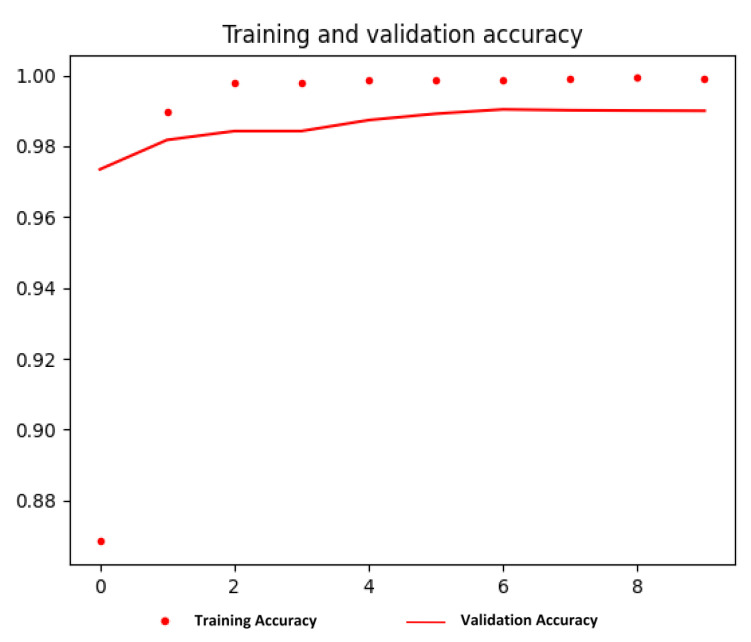
Training and validation accuracy for VGG16 model for VLR (classification) task. The *x*-axis represents the accuracy, and the *y*-axis represents the number of epochs. Also, the dotted line represents the training accuracy, and the solid line represents the validation accuracy.

The performance of the VGG16 model for the VLR (classification) task was monitored and validated during the training process using an accuracy matrix. The training and validation accuracy were visualised using the matplotlib library provided by the Python programming language, as depicted in Figure 6.

According to the above figure, the training and validation accuracy gradually increase and tend to coincide. The training accuracy was 0.9991, and the validation accuracy was 0.99. Moreover, the training loss and validation loss were 0.0041 and 0.0604, respectively. The training loss indicates that the model fits the training dataset, converges rapidly after epoch 3 and remains stable. Also, the validation loss indicates that the model fits the new dataset (i.e., the model is generalisable).

Moreover, the training accuracy is higher than the validation accuracy due to overfitting, despite the methods that have been applied to reduce it, as we mentioned earlier in the methodology section, which is summarised as follows:

1. Data augmentation.
2. Normalisation.
3. Dropout regularisation.

### 5.3. System Evaluation

**Figure 7:**
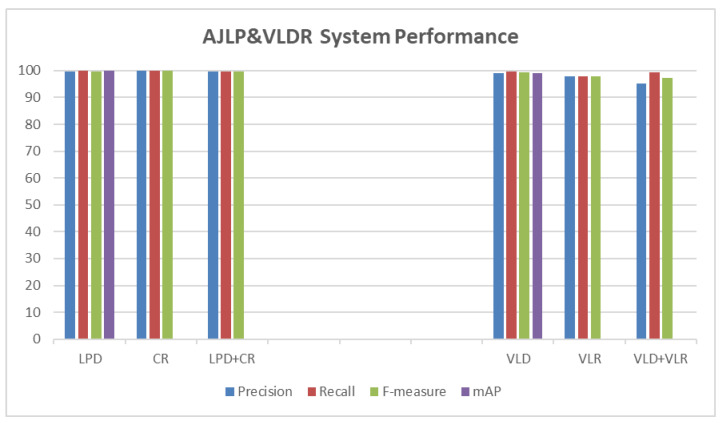
Performance of AJLP and VLDR stages on test set.

Several metrics have been conducted to evaluate the performance of the proposed approach. These matrices are precision, recall, and F-measure, which were applied to all test datasets. Moreover, the mAP measure was used to evaluate the YOLOv3 model of the detection tasks (i.e., LPD and VLD). In other words, each stage in the developed system was evaluated by calculating the test metrics for its dataset. Table 3 and Figure 7 show the performance of the AJLP and VLDR stages on the test set.

#### 5.3.1. License Plate Recognition

As described in the methodology chapter, the LPR stage consists of two other sub-stages: LPD and CR. Therefore, the effectiveness and robustness of this stage in Jordanian license plate recognition were evaluated by evaluating each sub-stage separately. The overall performance is then assessed by evaluating the two sub-stages together as a sequence.

##### License Plate Detection Evaluation

The performance of the YOLOv3 model that was trained to fit the Jordanian LPD task was evaluated using the previously mentioned text measures (metrics), which were defined for this task as follows [16]:

(1) $$Precision=\frac{TP}{TP+FP}$$

(2) $$Recall=\frac{TP}{TP+FN}$$

(3) $$F–measure=\frac{2*Precision*Recall}{Precision+Recall}\;\;\;\;\;0\leq F–measure\leq 1$$

where TP is the abbreviation of true positive, which indicates the number of correctly detected LPs, FP is the abbreviation of false positive, which indicates any part of the vehicle other than the LP that is incorrectly detected as LP, and FN is the abbreviation of false negative, which indicates LP incorrectly not detected. In other words, TP+FP is the total number of LPs positively detected, and TP+FN is the total number of ground truths (vehicles). The precision and recall were 99.6% and 100%, respectively. Meanwhile, the F-measure reached 99.8%. Also, the mAP was 99.9%.

##### Character Detection and Recognition Evaluation

The performance of the YOLOv3 model that was trained to fit the Jordanian LP character detection and recognition task was evaluated using the previously mentioned text measures (metrics) defined for this task. Moreover, the recognition result is valid when all LP characters are recognised correctly.

For this task, TP indicates the number of correctly recognised LPs, FP indicates an incorrectly recognised LP, and FN indicates an LP incorrectly recognised. In other words, TP+FP is the total number of LPs positively recognised, and TP+FN is the total number of ground truths (LPs). The precision and recall were 100% and 99.9%, respectively. Meanwhile, the F-measure reached 99.95%.

##### Overall License Plate Recognition Evaluation

The overall performance of the developed system for Jordanian license plate recognition was evaluated by evaluating the two sub-stages (i.e., LPD and character detection and recognition) together as a sequence. The test set from the first dataset was used for evaluation. For this task, TP indicates the number of correctly recognised LPs, FP indicates any part of the vehicle other than the LP that is incorrectly detected as LP, and FN indicates LP incorrectly not detected. In other words, TP+FP is the total number of LPs positively detected, and TP+FN is the total number of ground truths (vehicles) [90]. The precision and recall were 99.8% and 99.8%, respectively. Meanwhile, the F-measure reached 99.8%.

#### 5.3.2. Vehicle Logo Recognition

As described in the methodology chapter, the VLR stage consists of two other sub-stages: VLD and VLR (classification). Therefore, the effectiveness and robustness of this stage in vehicle recognition were evaluated by evaluating each sub-stage separately. The overall performance is then evaluated by evaluating the two sub-stages together as a sequence.

##### Vehicle Logo Detection Evaluation

The performance of the YOLOv3 model that was trained to fit the vehicle logos detection task was evaluated using the previously mentioned text measures. For this task, TP indicates the number of correctly detected VLs, FP indicates any part of the vehicle other than the VL that is incorrectly detected as VL, and FN indicates VL incorrectly not detected. In other words, TP+FP is the total number of VLs positively detected, and TP+FN is the total number of ground truths (vehicles). The precision and recall were 99% and 99.6%, respectively. Meanwhile, the F-measure reached 99.3%. Also, the mAP was 99.1%.

##### Vehicle Logo Recognition (Classification) Evaluation

The performance of the VGG16 model that was trained to fit the vehicle logos recognition (classification) task was evaluated using the previously mentioned text measures. At first, these metrics were calculated for each class (VL), which were defined as follows:

(4) $$Precision_{class\;i}=\frac{TP_{class\;i}}{TP_{class\;i}+FP_{class\;i}}$$

(5) $$Recall_{class\;i}=\frac{TP_{class\;i}}{TP_{class\;i}+FN_{class\;i}\;}$$

where class i refers to the VLs mentioned in Section 4, $TP_{class\;i}$ indicates the number of correctly recognised (classified) VLs for class i, $FP_{class\;i}\;$ indicates an incorrectly recognised VL for class i, and $FN_{class\;i}$ indicates VL incorrectly not recognised for class i. In other words, $TP_{class\;i}+FP_{class\;i}$ is the total number of VLs positively recognised and $TP_{class\;i}+FN_{class\;i}$ is the total number of ground truths. After that, the micro-average precision and micro-average recall were calculated as follows [91,92]:

(6) $$\;Micro–Average\;Precision=\frac{\sum_{i=1}^{n}TP_{class\;i}}{\sum_{i=1}^{n}TP_{class\;i}+\sum_{i=1}^{n}FP_{class\;i}}$$

(7) $$Micro–Average\;Recall=\frac{\sum_{i=1}^{n}TP_{class\;i}}{\sum_{i=1}^{n}TP_{class\;i}+\sum_{i=1}^{n}FN_{class\;i}}$$

where n is the number of classes. After that, the micro F-measure based on average precision and recall was calculated as follows [91,92]:

(8) $$Micro\;\;F–measure=\frac{2*Micro-Average\;Preccision*Micro-Average\;Recall}{Micro-Average\;Precision+Micro-Average\;Recall}$$

The micro-average precision and micro-average recall were 98% and 98%, respectively. Meanwhile, the micro F-measure reached 98%.

##### Overall Vehicle Logo Recognition Evaluation

The overall performance of the developed system for VLR was evaluated by evaluating the two sub-stages (i.e., VLD and VLR (classification)) together as a sequence. The test set from the first dataset was used for evaluation. TP indicates the number of correctly recognised VLs, FP indicates any part of the vehicle other than the VL that is incorrectly detected as VL, and FN indicates VL incorrectly not detected. In other words, TP+FP is the total number of VLs positively detected, and TP+FN is the total number of ground truths (vehicles). The precision and recall were 95.3% and 99.5%, respectively. Meanwhile, the F-measure reached 97.4%.

### 5.4. Discussion

The proposed AJLP and VLDR system achieved high performance in text measures (i.e., precision, recall, and F-measure) and mAP measures for detection tasks, as shown in Table 3 and Figure 7, respectively. For LPD, the precision, recall, F-measure, and mAP were 99.6%, 100%, 99.8%, and 99.9%, respectively. While for character recognition, the precision, recall, and F-measure were 100%, 99.9%, and 99.95%, respectively. The performance of the LPR stage was evaluated by evaluating these two sub-stages as a sequence, where the precision, recall, and F-measure were 99.8%, 99.8%, and 99.8%, respectively. Furthermore, for VLD, the precision, recall, F-measure, and mAP were 99%, 99.6%, 99.3%, and 99.1%, respectively, while for VLR, the precision, recall, and F-measure were 98%, 98%, and 98%, respectively. The performance of the VLR stage was evaluated by evaluating these two sub-stages as a sequence, where the precision, recall, and F-measure were 95.3%, 99.5%, and 97.4%, respectively. But there are some cases where our system went wrong. For the LPD task, the LP is not detected for some vehicles. For the task of character detection and recognition, there are some vehicles whose LP characters are not all recognised, and in other cases, in some vehicles, the characters have been incorrectly recognised. For both stages together (i.e., LPD and character detection and recognition), in some vehicles, the LP was not recognised due to being undetected, while in others, the LP was detected but incorrectly recognised. For the VLD task, the logo of some vehicles was not detected, while in others, the logo was not present but was detected as a logo. For the VLR task, some VLs were incorrectly recognised. For both stages together (i.e., VLD and VLR), on some vehicles, the logo was detected but incorrectly recognised; in other cases, the logo was not recognised because it was not detected; and on some vehicles, the logo was not present, but it was detected as a logo and incorrectly recognised.

Moreover, the ALP and VLDR system can generally be applied for still images and video streams. Also, it can be applied to images in PNG or JPG formats. Further, in object detection tasks, the true negative from the confusion matrix represents all predicted bounding boxes that do not include objects (i.e., a corrected misdetection). This case is not useful for object detection because there are a lot of potential true negatives in the image that should not be detected [93]. Our research applies the proposed algorithm for still images from the collected dataset. Also, it will be applied to images in JPG format. Moreover, the proposed algorithm will be applied to Jordanian vehicles only. Further, in our Jordanian vehicle dataset, all vehicles have LPs. So, the true negative metric was ignored.

## 6. Conclusions

In this research, an efficient ALP and VLDR system for Jordanian vehicles has been developed. As mentioned earlier, the AJLP and VLDR system mainly consists of two stages: LPR and VLR. Moreover, these two main stages also consist of two other sub-stages. The first stage is LPD and character detection and recognition, while the second stage is VLD and VLR (classification). DL-based CNN using TL was used to accomplish the proposed system. The YOLOv3 model was re-trained using TL to accomplish the LPD, character detection and recognition, and VLD tasks. In contrast, the VGG16 model was also re-trained to accomplish the VLR (classification) task. Since these sub-stages have different tasks, four datasets were collected for training and testing. The first dataset consists of 7035 Jordanian vehicle images used to train and test the YOLOv3 model for the Jordanian LPD task. The second dataset consists of 7176 Jordanian LPs used to train and test the YOLOv3 model for CR of Jordanian LPs. The third dataset consists of 8271 Jordanian vehicle images used to train and test the YOLOv3 model for the VLD task. The fourth dataset consists of 158,230 VL images used to train and test the VGG16 model for the VLR task. The implemented experimental results proved a high performance of our proposed system in terms of precision, recall, F-measure, and mAP. Moreover, the experiment showed that the training accuracy is higher than the validation accuracy of the VGG16 model due to overfitting.

As a future work, we will develop a system to detect and recognise Jordanian LPs and VLs from a real-time video stream. Moreover, we will expand the VL dataset to include new VLs. Also, this research can be extended to compare with other SOTA methods and to apply other techniques to reduce overfitting.

## Figures and Tables

**Table 1:** Datasets partitioning.

Stage	Training Dataset	Validation Dataset	Testing Dataset
LPD	70%	15%	15%
Character Detection and Recognition	70%	15%	15%
VLD	70%	15%	15%
VLR	70%	20%	10%

**Table 2:** Input size and hyper-parameter configuration.

Stage	CNN Model	Input Size	Batch Size	Number of Epochs
LPD	YOLOv3	416	32	100
Character Detection and Recognition	YOLOv3	416	32	100
VLD	YOLOv3	416	32	100
VLR	VGG16	224	32	10

**Table 3:** Performance of AJLP and VLDR stages on test set.

Stage	Precision	Recall	F-Measure	mAP
LPD	99.6%	100%	99.8%	99.9%
Character Detection and Recognition	100%	99.9%	99.95%	____
Overall License Plate Recognition	99.8%	99.8%	99.8%	____
Vehicle Logo Detection	99%	99.6%	99.3%	99.1%
Vehicle Logo Recognition (Classification)	98%	98%	98%	____
Overall Vehicle Logo Recognition	95.3%	99.5%	97.4%	____

## Data Availability

The dataset will be available for training and testing only to prevent misuse and privacy breaches.
